# Modulation of trigeminovascular activity by leptin: a novel antinociceptive mechanism?

**DOI:** 10.1186/1129-2377-14-S1-P76

**Published:** 2013-02-21

**Authors:** M Martins-Oliveira, J Hoffmann, S Akerman, PJ Goadsby

**Affiliations:** 1 Headache Group, Department of Neurology, University of California San Francisco, San Francisco CA, USA

## Introduction

Fasting is a recognized trigger of headache in susceptible individuals. Leptin is a peptide hormone encoded by the mouse obese gene (ob)[1] and is mainly secreted by white adipocytes. Plasma levels of leptin are regulated to reflect body energy stores: levels of leptin fall in response to fasting and are increased in several models of murine obesity[2]. Leptin signaling decreases feeding and increases energy expenditure by activating the longform of its receptor (LepRb) in areas of the brain[3] including the brainstem, midbrain, hypothalamus, thalamus and cortex[4].

## Aim

To study the effect of acute administration of leptin on activity in the trigeminocervical complex (TCC) in response to middle meningeal artery (MMA) stimulation.

## Methods

Adult male Sprague-Dawley rats were anesthetized with pentobarbitone (60 mgkg-1) and cannulated for measurement of blood pressure and intravenous administration of supplementary anesthesia with propofol (15-20 mgkg-1hr-1). The parietal bone was removed over the MMA for electrical stimulation of the dura mater and electrophysiological recording of second order neurons in the TCC using a tungsten electrode. Rats received either rat recombinant leptin in a dosage of 1 mgkg-1 (i.v.) dissolved in sterile water, or sterile water alone as the control group. The effects of leptin on TCC activity in response to MMA stimulation were recorded.

## Results

Leptin significantly reduced cell firing in response to trigeminovascular activation within the TCC (p < 0.05). This effect reached a maximum inhibition of nearly 12% at 45 minutes post-infusion of leptin. Infusion of sterile water alone had no significant effect on MMA stimulation-evoked firing.

## Conclusion

The data show leptin treatment inhibits stimulus-evoked trigeminal activity, which might explain, in part, why fasting, a condition characterized by low levels of circulating leptin, may have a role in headache. Exploring the interaction between feeding physiology and trigeminovascular nociceptive mechanisms may contribute to our understanding of this very common trigger.
